# ASO Author Reflections: Resection Margins in Distal Pancreatectomy for Ductal Adenocarcinoma—Does Surgery Tell the Whole Story?

**DOI:** 10.1245/s10434-021-10515-y

**Published:** 2021-07-24

**Authors:** Mushegh A. Sahakyan, Knut Jørgen Labori, Bjørn Edwin

**Affiliations:** 1 The Intervention Centre, Rikshospitalet, Oslo University Hospital, Oslo, Norway; 2 Division of Emergencies and Critical Care, Department of Research and Development, Oslo University Hospital, Oslo, Norway; 3 Department of Surgery N1, Yerevan State Medical University, Yerevan, Armenia; 4 Institute of Clinical Medicine, University of Oslo, Oslo, Norway; 5 Department of Hepato-Pancreato-Biliary Surgery, Rikshospitalet, Oslo University Hospital, Oslo, Norway

## Past

Resection margins are considered to be one of the few surgeon-controlled parameters affecting prognosis in pancreatic ductal adenocarcinoma (PDAC). Distal pancreatectomy (DP) is the standard procedure for PDAC in the distal pancreas, and different surgical approaches have been suggested to increase its radicality and avoid positive resection margins (R1).^1^,^2^ At the same time, the meticulousness of the pathology work-up was shown to affect the R1 rate.^3^ Pathology examination protocols for DP specimens have only recently been published, and there is currently no international consensus.^4^ As a result, little is known about resection margin status and its clinical relevance in DP for PDAC.

## Present

This study from a tertiary referral center for pancreatic surgery examined the R-status in DP specimens with PDAC, focusing on standardization and meticulousness of the pathology work-up.^5^ The introduction of a standardized grossing protocol with emphasis on extensive tissue sampling resulted in an increase in R1-rate from 60.4 to 83.1% (*p* = 0.005). Furthermore, it was independently associated with R1 in the multivariable model. Since neither surgeons/surgical technique nor the indications for DP in PDAC changed during the study period, the increase in R1-rate should be attributed to the pathology examination rather than to any aspect of surgery. Hence, the reported R1-rate seems to reflect the real-life situation in a high-volume center where DP for cancer is regularly performed and a standardized meticulous pathology protocol is systematically used.

Positive anterior pancreatic surface, but not R1 itself, was found to be a significant prognostic factor for overall survival. To the best of the authors’ knowledge, this is the first study to report about such an impact of the anterior pancreatic surface in patients undergoing DP for PDAC. Nevertheless, one should remember that the anterior pancreatic surface is unaffected by the extent of surgery and its involvement is rather determined by the location of the tumor.

## Future

Considering the prognostic implications of the anterior pancreatic surface, specific reporting of its involvement rather than including it in an overall R1-status without further specification seems reasonable. Furthermore, neoadjuvant chemo- and/or radiotherapy may be considered when involvement of the anterior pancreatic surface is suggested on preoperative imaging.

Overall, widespread implementation of standardized pathology examination protocol for DP specimens is essential. This will allow for not only obtaining robust evidence from cancer registries and randomized trials but also drawing valid comparisons between literature data. Most importantly, though, it will reveal the real prognostic impact of R1 in these patients and help surgical community to understand the extent of oncological benefits expected from various surgical approaches in DP.
